# Safety of COVID-19 vaccine in patients with myasthenia gravis: a self-controlled case series study

**DOI:** 10.3389/fimmu.2023.1141983

**Published:** 2023-05-08

**Authors:** Zhe Ruan, Xiao Huan, Yue Su, Yong-Lan Tang, Dong-Dong Meng, Da-Lin Ren, Chun-Hong Li, Si-Jia Hao, Chong-Bo Zhao, Su-Shan Luo, Zhu-Yi Li, Ting Chang

**Affiliations:** ^1^Department of Neurology, Tangdu Hospital, The Fourth Military Medical University, Xi’an, China; ^2^Huashan Rare Disease Center, Department of Neurology, Huashan Hospital Fudan University, Shanghai, China; ^3^Department of Experimental Surgery, Tangdu Hospital, The Fourth Military Medical University, Xi’an, China

**Keywords:** myasthenia gravis, COVID-19 vaccines, vaccine hesitancy, self-control study, vaccine safety

## Abstract

**Background:**

The safety of COVID-19 vaccines has been clarified in clinical trials; however, some immunocompromised patients, such as myasthenia gravis (MG) patients, are still hesitant to receive vaccines. Whether COVID-19 vaccination increases the risk of disease worsening in these patients remains unknown. This study aims to evaluate the risk of disease exacerbation in COVID-19-vaccinated MG patients.

**Methods:**

The data in this study were collected from the MG database at Tangdu Hospital, the Fourth Military Medical University, and the Tertiary Referral Diagnostic Center at Huashan Hospital, Fudan University, from 1 April 2022 to 31 October 2022. A self-controlled case series method was applied, and the incidence rate ratios were calculated in the prespecified risk period using conditional Poisson regression.

**Results:**

Inactivated COVID-19 vaccines did not increase the risk of disease exacerbation in MG patients with stable disease status. A few patients experienced transient disease worsening, but the symptoms were mild. It is noted that more attention should be paid to thymoma-related MG, especially within 1 week after COVID-19 vaccination.

**Conclusion:**

COVID-19 vaccination has no long-term impact on MG relapse.

## 1 Introduction

Coronavirus disease 2019 (COVID-19) has spread rapidly worldwide. Since its outbreak in 2020, this devastating viral infection has infected approximately 630 million people, with total deaths of over 6.5 million. Rapid delivery of safe and effective vaccines is the most promising strategy to control the COVID-19 pandemic. There is a global consensus concerning COVID-19 vaccination. Since the first mass vaccination program started in December 2020, approximately 64% of the population worldwide has received two doses of the COVID-19 vaccine (updated to 30 October 2022: Our World in Data: https://ourworldindata.org). Although the safety of COVID-19 vaccines has also been clarified in clinical trials, vaccine hesitancy still exists in some immunocompromised patients, such as patients with myasthenia gravis (MG) (1, 2). The main reason for vaccine hesitancy in these immunocompromised patients was fear of vaccine-related symptom exacerbation.

MG is a prototypical autoimmune disorder caused by specific autoantibodies at the neuromuscular junction, in which CD4^+^ T cells and B cells play important roles. Vaccines were shown to elicit a strong humoral response by production of neutralizing antibodies, as well as a strong cellular response by inducing functional and pro-inflammatory CD4+ and CD8+ T cells and expression of Th1 cytokines, theoretically worsening symptoms in patients with autoimmune diseases (3). Given that most patients with MG are on immunosuppressive or immunomodulatory therapies, there is also a theoretical concern that MG patients may be at greater risk of infection or even severe manifestations of COVID-19 than healthy people. Thus, they should be prioritized for vaccination against COVID-19. However, there are no randomized controlled trials to confirm the safety of the COVID-19 vaccine in MG patients to date.

Controversial reports exist among the available studies. Autoimmune disease exacerbation or new-onset autoimmune disease has been described following COVID-19 vaccination (4, 5). For example, Watad et al. reported that two patients with MG developed a myasthenic crisis after vaccination and underwent mechanical ventilation (6), while in several subsequent studies, COVID-19 vaccines were confirmed to be safe in patients with MG (7–10). Only a few vaccine-related exacerbations were reported, but the symptoms were very mild and did not need additional treatment. Owing to the single-arm design and lack of control, these studies did not determine the risk of disease exacerbation in unvaccinated MG patients. Thus, whether COVID-19 vaccination increases the risk of symptom exacerbation in MG patients remains unknown.

In this study, we collected data from two registered cohorts in China. We adopted the self-controlled case series (SCCS) method to evaluate the risk of disease exacerbation in COVID-19-vaccinated MG patients.

## 2 Materials and methods

### 2.1 Study design

This multi-center retrospective cohort study used the SCCS method. SCCS was originally developed to evaluate the association of rare adverse events with vaccination (11–13), with the participants in the cohort serving as their own controls. Any covariate that does not change over time will be controlled, so this design has an advantage in analyzing vaccine-related studies, even with small sample sizes (14, 15).

### 2.2 Exposure period settings

**Figure 1:**
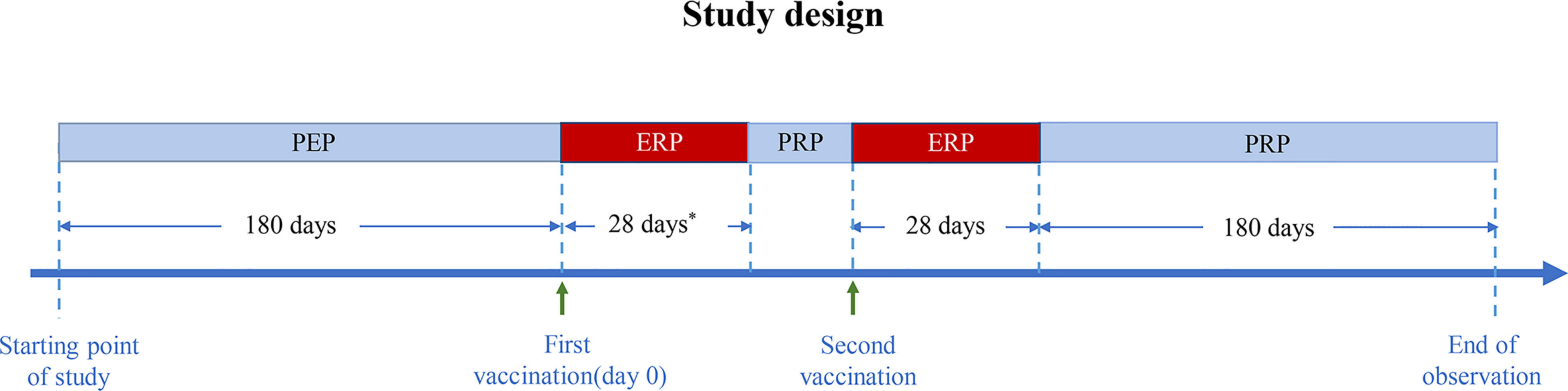
Diagram of the study design. PEP, pre-exposure risk period; ERP, exposure risk period; PRP, post-risk period. * Minimum interval between two doses: 21 days.

The pre-exposure risk period (PEP) was defined as 180 days preceding the first vaccination, and the exposure risk period (ERP) was defined as 28 days after the first vaccination. If the patient received two doses of COVID-19 vaccines, the ERP was defined as 28 days after the first dose plus 28 days after the second dose. Because the minimum interval between two doses is 21 days, the ERP ranged from 49 to 56 days. The post-risk period (PRP) was defined as follows: if the patient received two COVID-19 vaccine doses and the interval between them was over 28 days, the PRP consisted of two periods: the first period was from 29 days after the first COVID-19 vaccination to the second vaccination, and the second period was from 29 to 209 days after the second vaccination. The study design flow diagram is shown in **Figure 1**.

### 2.3 Data source

The data in this study were collected from the MG database at Tangdu Hospital, the Fourth Military Medical University, and the Tertiary Referral Diagnostic Center at Huashan Hospital, Fudan University, from 1 April 2022 to 31 October 2022. Both databases used previously published structured data collection forms to record longitudinal data on MG patients (16–21).

### 2.4 Inclusion and exclusion criteria

The inclusion criteria were that the patients with MG were ≥18 years old and had received at least one dose of the COVID-19 vaccine. The diagnosis of MG was based on the fluctuating skeletal muscle weakness and at least one of the following three conditions: (1) neostigmine testing was positive; (2) seropositive antibodies for acetylcholine receptor (AChR), muscle-specific kinase (MuSK), or lipoprotein-related protein 4; and (3) abnormal repetitive nerve stimulation (RNS) (3 Hz RNS was applied to the facial, ulnar, axillary, and accessory nerves, and the amplitude of the compound muscle action was more than 10% decreased).

Patients who met one or more of the following exclusion criteria were excluded: (1) the patient experienced disease onset after COVID-19 vaccination; (2) the disease course was less than 180 days before vaccination; (3) the patient underwent thymectomy during the observation period; and (4) crucial information was missing, including disease exacerbation or COVID-19 vaccination information.

### 2.5 Data collection

An electronic questionnaire was sent to the participants *via the* online chat program WeChat, which researchers developed before the formal study. The MG patients who registered in the database and had detailed contact were enrolled in our study. Patients who could not operate the software completed the questionnaire with the help of their caretakers. The questionnaire included basic demographic characteristics; the COVID-19 vaccination information was assessed by a single question: “Have you ever received the COVID-19 vaccine?” Of the questionnaires returned, all vaccinated MG patients were screened. Baseline characteristics and all available follow-up information from the observation periods were retrieved from the database. All vaccinated MG patients were followed up face-to-face or by phone to confirm disease exacerbation during the observation periods. COVID-19 vaccine-related information was obtained from the electronic vaccination card, including vaccination time and type.

The data were systematically collected using a predefined form. Basic demographic characteristics included sex and age. Clinical characteristics included the following: disease onset, disease duration (defined as the period from disease onset to vaccination), thymic status, thymectomy status, serological profile, the Myasthenia Gravis Foundation of America Task Force Post-Intervention Status (MGFA-PIS) at the first vaccination and study end, and treatment regimen at the time of the first vaccination. The immunosuppression treatments were defined as having received steroids and non-steroid immunosuppressants. The non-steroid immunosuppressants include tacrolimus, mycophenolate mofetil, azathioprine, and rituximab. The treatment duration was at least 1 month prior to vaccination for patients undergoing steroid treatment and at least 3 months prior to vaccination for those undergoing non-steroid immunosuppressant treatment. The COVID-19 vaccination information included the vaccine profile, vaccination date, and vaccine-related side effects.

### 2.6 Outcome measure

The primary outcome was the number of disease exacerbations during observation periods. Disease exacerbation was defined as the new onset or worsening of pre-existing muscle weakness reported by the patient or recorded in the database. The investigator re-evaluated the MG Activity of Daily Living (MG-ADL) score for patient-self-reported exacerbation, and records were reviewed to prevent recall bias.

### 2.7 Statistical analyses

Descriptive statistics were used to analyze the baseline data. Categorical variables were presented as counts and percentages; continuous variables following normal distribution were presented as the mean and standard deviation (SD) and otherwise as the median and interquartile range (IQR). We performed the analyses using conditional Poisson regression. Models were checked for overdispersion by the R software “qcc” package; *p* < 0.05 indicated overdispersion, in which condition quasi-Poisson regression was an alternative. An offset for the risk period length was included in the model. The incidence rate ratio (IRR) of exacerbation in the ERP relative to the PEP, in the PRP relative to the PEP, and their 95% confidence intervals (CIs) were estimated using the regression model.

### 2.8 Sensitivity analyses

The observation period after the first vaccination was divided into three periods: 0–7 days, 8–14 days, and 15–21 days. To assess the risk of disease exacerbation after vaccination over time, the IRR relative to the PEP was calculated for each of the three periods. We performed subgroup analyses for different covariates, including age (classified as <50 or ≥50 years), disease duration (classified by median disease duration of <49 or ≥49 months), sex, thymic status, and thymectomy status. Sensitivity analyses were performed to check for heterogeneity within each subgroup, and the likelihood ratio test was used to assess the interaction effect.

## 3 Results

**Figure 2:**
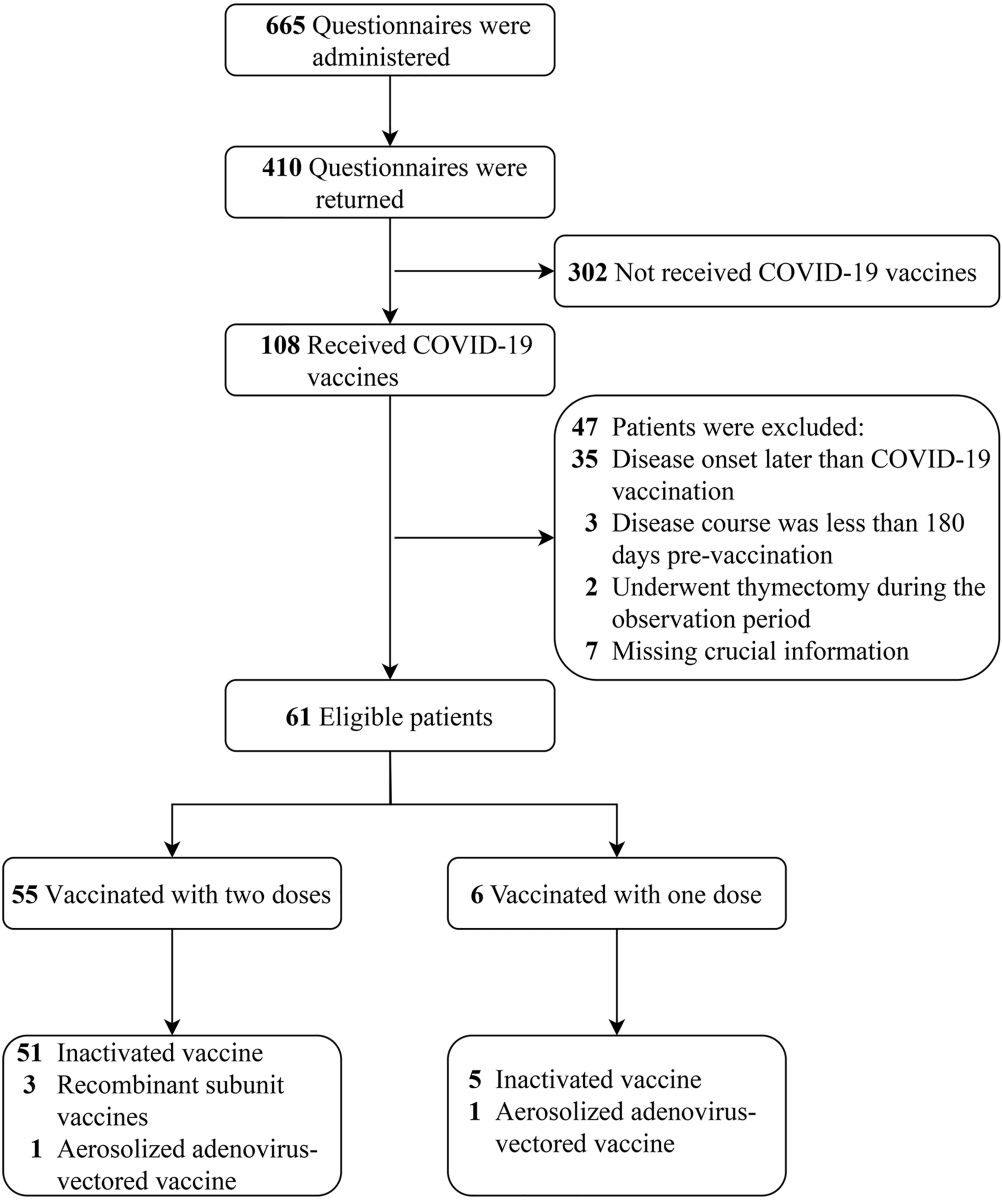
The selection flowchart for patients with myasthenia gravis.

A total of 665 electronic questionnaires were administered, and 410 were returned; the questionnaire recovery rate was 61.7%. Of these patients, only 108 (26.3%) received the COVID-19 vaccine. Forty-seven patients were excluded after meeting exclusion criteria, and 61 patients were included in the final analysis. The patient selection process is shown in **Figure 2**.

**Table 1** shows the demographic and clinical characteristics. A total of 61 patients were included; 30 (49.2%) were female, and the mean age was 45.64 (SD ± 13.48) years. Fifty-six (91.8%) patients were positive for anti-AChR, four (6.6%) patients were positive for anti-MuSK, and one (1.6%) patient was seronegative. A total of 45 (73.8%) patients were administered immunosuppression at the time of the first vaccination. Only one patient was given additional immunosuppressants during the ERP because of disease exacerbation; the other patients maintained their pre-vaccination treatment regimen. Fifty-five (90.2%) patients received two vaccine doses, 6 (9.8%) patients received one dose of vaccine, and 56 (91.8%) patients received the inactivated vaccine at the first vaccination. According to the MGFA-PIS (22), 43 (70.5%) patients reached minimal manifestation status (MMS) or better at the end of the study.

**Table 1:** The demographic and clinical characteristics of the study participants.

Characteristics	Total (*n* = 61)
Sex, *n* (%)	
Female	30 (49.2)
Male	31 (50.8)
Age, years, mean (SD)	45.64 (13.48)
Disease course, months, median [IQR]	49.00 [27.00, 76.00]
Thymic status, *n* (%)	
Non-thymoma	40 (65.6)
Thymoma	21 (34.4)
Thymectomy, *n* (%)	
No	38 (62.3)
Yes	23 (37.7)
Serological profile, *n* (%)	
AChR+	56 (91.8)
MuSK+	4 (6.6)
SNMG	1 (1.6)
MGFA-PIS at the first vaccination, *n* (%)	
MMS or better	42 (68.9)
Improved	16 (26.2)
Unchanged	3 (4.9)
Treatment regimen at the first vaccination, *n* (%)	
Non-immunosuppression	16 (26.2)
Immunosuppression^†^	45 (73.8)
Number of doses, *n* (%)	
One dose	6 (9.8)
Two doses	55 (90.2)
Vaccine profile (first dose), *n* (%)	
Inactivated vaccine	56 (91.8)
Recombinant subunit vaccine	3 (4.9)
Aerosolized adenovirus-vectored vaccine	2 (3.3)
Vaccine profile (second dose), *n* (%)	
Inactivated vaccine	51/55 (92.7)
Recombinant subunit vaccine	3/55 (5.5)
Aerosolized adenovirus-vectored vaccine	1/55 (1.8)
Interval between two doses, days, mean (SD)	35.11 (27.32)
Exposure risk period, days, mean (SD)	48.18 (10.34)
Post-exposure risk period, days, mean (SD)	188.38 (25.21)
MGFA-PIS at the end of the study, *n* (%)	
MMS or better	43 (70.5)
Improved	17 (27.9)
Unchanged	1 (1.6)

SD, standard deviation; IQR, interquartile range; AChR+, seropositivity for acetylcholine receptor antibodies; MuSK+, seropositivity for muscle specific tyrosine kinase antibodies; SNMG, seronegative myasthenia gravis; MGFA, Myasthenia Gravis Foundation of America; PIS, post-intervention status; MMS, minimal manifestation status; d, day.

^†^Treatment with prednisone, tacrolimus, mycophenolate mofetil, azathioprine, and rituximab.

In the PEP, nine patients experienced 10 disease exacerbations, while in the ERP, six were observed in five patients, of whom one patient experienced two disease exacerbations, both on the day after COVID-19 vaccination. In the PRP, 10 disease exacerbations were observed in nine patients. All disease exacerbations during the ERP were mild, the average MG-ADL increased by 2.2 points, and no patients required hospitalization. Generally, the symptoms were self-limited and resolved within 7 days without additional treatment. The symptoms persisted in only one patient, and the symptoms were completely relieved within 1 month after being given low-dose steroids (0.5 mg/kg). The follow-up was recorded in the database to confirm this result. **Table 2** shows the clinical characteristics of patients with disease exacerbation.

**Table 2:** Details on the patients with exacerbation during the pre-exposure risk period, the exposure risk period, and the post-risk period.

ID	Sex	Age	Serological profile	Thymic status	Thymectomy	MGFA-PIS at the first vaccination	Vaccine profile	Number of exacerbations
Pre-exposure risk period								
11	M	55	AChR+	Non-thymoma	No	CSR	Inactivated vaccine	1
15	F	47	AChR+	Thymoma	Yes	MMS	Inactivated vaccine	1
20	M	51	AChR+	Non-thymoma	No	I	Recombinant subunit vaccine	1
22	M	59	AChR+	Non-thymoma	No	I	Inactivated vaccine	1
25	M	33	AChR+	Thymoma	Yes	I	Inactivated vaccine	1
31	M	53	AChR+	Non-thymoma	No	PR	Inactivated vaccine	1
42	F	22	MuSK+	Non-thymoma	No	I	Inactivated vaccine	1
50	F	51	AChR+	Non-thymoma	No	I	Inactivated vaccine	2
51	F	50	AChR+	Thymoma	Yes	PR	Inactivated vaccine	1
Exposure risk period								
3	M	53	AChR+	Thymoma	Yes	MMS	Inactivated vaccine	1
13	F	24	AChR+	Non-thymoma	No	I	Inactivated vaccine	2
24	M	49	AChR+	Thymoma	Yes	PR	Inactivated vaccine	1
25	M	33	AChR+	Thymoma	Yes	I	Inactivated vaccine	1
47	F	32	MuSK+	Thymoma	No	CSR	Inactivated vaccine	1
Post-risk period								
6	F	21	AChR+	Non-thymoma	No	MMS	Inactivated vaccine	1
18	M	44	AChR+	Thymoma	Yes	MMS	Inactivated vaccine	1
21	M	73	AChR+	Non-thymoma	No	I	Inactivated vaccine	1
28	M	54	AChR+	Thymoma	Yes	I	Inactivated vaccine	2
29	M	64	AChR+	Non-thymoma	No	U	Inactivated vaccine	1
30	F	57	AChR+	Non-thymoma	No	PR	Inactivated vaccine	1
37	M	45	AChR+	Non-thymoma	No	PR	Inactivated vaccine	1
44	M	39	AChR+	Thymoma	Yes	I	Inactivated vaccine	1
48	F	65	AChR+	Non-thymoma	No	U	Inactivated vaccine	1

ID, identity document; M, male; F, female; AChR+, seropositivity for acetylcholine receptor antibodies; MuSK+, seropositivity for muscle specific tyrosine kinase antibodies; MGFA, Myasthenia Gravis Foundation of America; PIS, post-intervention status; MMS, minimal manifestation status; CSR, complete stable remission; PR, pharmacologic remission; I, improved; U, unchanged.

The IRR was estimated by conditional Poisson regression. Compared with the PEP, the rate of disease exacerbation in the ERP did not show a statistical difference (IRR: 2.24, 95% CI: 0.76–6.04, *p* = 0.12). In the PRP, the rate of disease exacerbation has no statistical significance when compared with the PEP (IRR: 0.96, 95% CI: 0.38–2.39, *p* = 0.92). In the sensitivity analysis, compared with the PEP, the IRR was 7.71 (95% CI: 1.73–25.22, *p* = 0.002) within 7 days after the first vaccination. For 8–14 days and 15–21 days, the IRRs were 2.57 (95% CI: 0.14–13.42, *p* = 0.37). **Table 3** presents the IRR (95% CI) for the primary outcome during the prespecified risk periods.

**Table 3:** Incidence rate ratios of the exacerbations during the exposure risk period, the post-risk period, and the observation period after the first vaccination.

Period	Number of exacerbations	IRR (95% CI)	*p*-value
PEP (control period)	10	1 (ref)	
ERP	6	2.24 (0.76–6.04)	0.12
PRP	10	0.96 (0.38–2.39)	0.92
0 to 7 days after the first vaccination	3	7.71 (1.73–25.22)	0.002
8 to 14 days after the first vaccination	0	2.57 (0.14–13.42)	0.37
15 to 21 days after the first vaccination	0	2.57 (0.14–13.42)	0.37

IRR, incidence rate ratios; CI, confidence interval; PEP, pre-exposure risk period; ERP, exposure risk period; PRP, post-risk period.

### 3.1 Subgroup analyses

**Figure 3:**
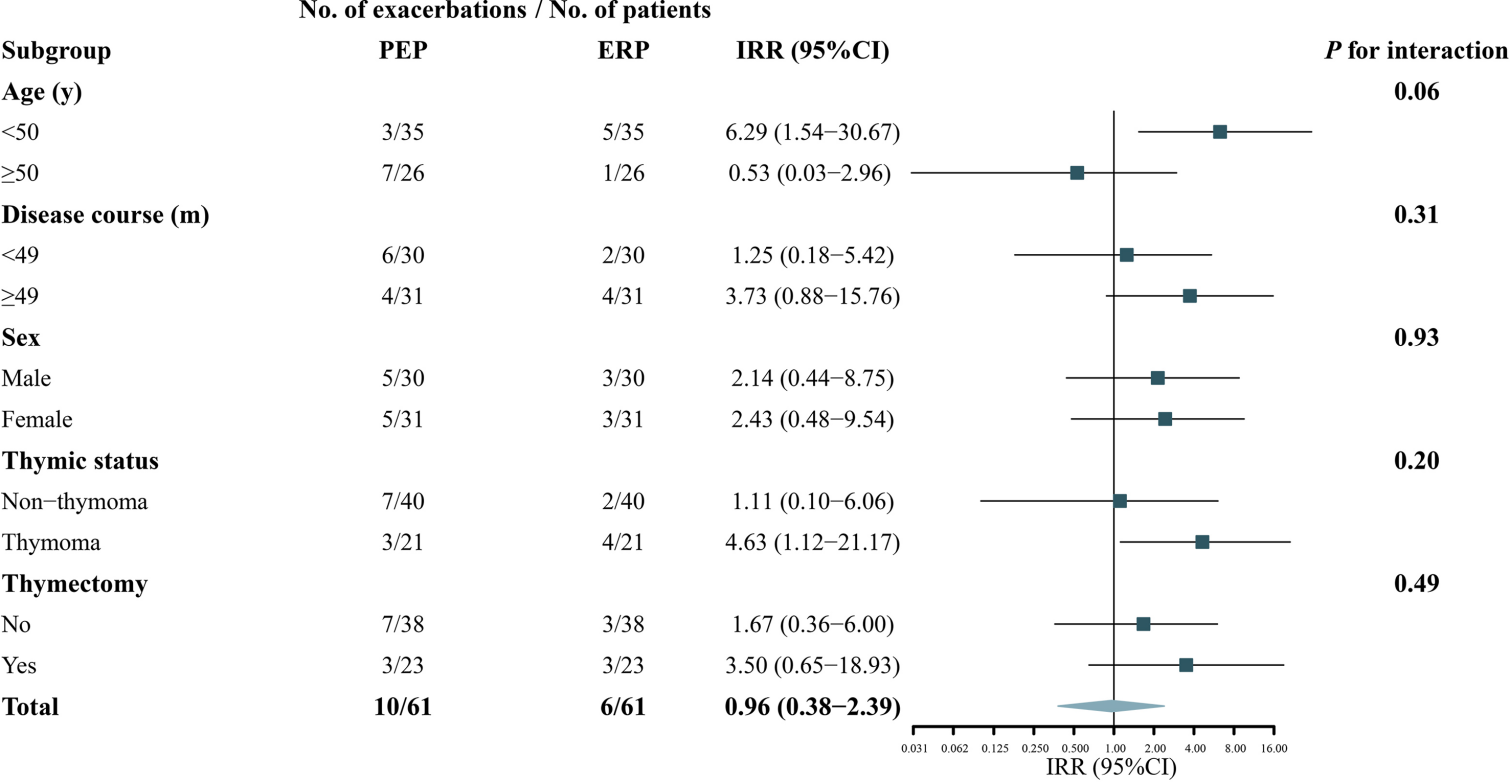
Subgroup analyses of the association between COVID-19 vaccination and exacerbation in patients with myasthenia gravis. A forest plot depicting the incidence rate ratios (IRR) of exacerbation in ERP relative to PEP in different subgroups. PEP, pre-exposure risk period; ERP, exposure risk period.

No heterogeneity was noted in the predefined subgroups, including for sex, thymectomy status, and disease course. In the age subgroup, the IRR difference was observed between the two subgroups (IRR: 6.29, 95% CI: 1.54–30.67 versus IRR: 0.53, 95% CI: 0.03–2.96), but the interaction effect test was not statistically significant (*p* = 0.06), meaning the difference was most likely derived from random errors. In addition, although the difference did not reach statistical significance, most of the patients with disease exacerbation in the ERP belonged to the thymoma-related MG group (*p* = 0.20). The IRRs of the subgroups are shown in **Figure 3**. **Table 4** shows the COVID-19 vaccine-related side effects reported in previous studies that resolved spontaneously without additional treatment.

**Table 4:** The vaccine-related side effects after COVID-19 vaccination.

Patient ID	Sex	Age^†^	Side effects	Duration of side effects (days)
First vaccination				
4	F	51	Focal symptoms^‡^	2
13	F	24	Fatigue	7
26	M	61	Focal symptoms; Fatigue	1
35	M	33	Focal symptoms; Fatigue	1
42	F	22	Focal symptoms	2
49	M	36	Focal symptoms	3
56	F	23	Focal symptoms; Fever	3
58	F	34	Focal symptoms	2
59	M	47	Focal symptoms; Fever	3
Second vaccination				
13	F	24	Fatigue	7
47	F	32	Focal symptoms; Fatigue	3
55	M	54	Focal symptoms	5
58	F	34	Focal symptoms	3

ID, identity document; M, male; F, female; d, day.

^†^ The age when the first dose was received.

^‡^ Pain, redness, and swelling at the injection site.

## 4 Discussion

We retrospectively reviewed two MG cohorts and evaluated the safety of the COVID-19 vaccine in patients with MG using the SCCS design. In our study, the exacerbation rates of MG were not increased during the observation period, consistent with previous studies. We performed sensitivity analyses and found that the IRR increased within 0–7 days after the first vaccination. Owing to the sample size limitation, the results needed to be taken with caution. All the disease exacerbations during the ERP were mild, and no patients required hospitalization. The COVID-19 vaccine-related side effects in patients with MG were comparable to those observed in the general population (23, 24).

In our study, only 25.7% of the 410 patients received the COVID-19 vaccine, while 89.2% of the total population of China (1.43 billion) have received at least one dose. To some extent, this disparity reflects the severe vaccine hesitancy among MG patients. Two survey studies describe the reasons for vaccine hesitancy in patients with autoimmune diseases: (1) fear of disease exacerbation following COVID-19 vaccination; (2) fear of drug–vaccine interactions. Despite the low prevalence of MG, China has a large population, resulting in a large number of MG patients; protecting these patients is critical to combating COVID-19. Several studies have reported the safety of the COVID-19 vaccine in MG patients, and MG exacerbation after vaccination was uncommon (7, 10, 25). However, because of the lack of a control group, the method in most of these studies was mainly descriptive and based on confidence intervals rather than hypothesis testing. Doron et al. used a controlled pre–post design and showed that both the MG incidence and exacerbation rate were consistent with previous reports, but they mainly focused on the BNT162b2 mRNA COVID-19 vaccine; other vaccine types were not included (8).

Most of the patients in our study received inactivated vaccines. The IRR was not increased during the ERP, consistent with the results of Doron et al. (8). In the sensitivity analysis, we observed an interesting phenomenon: compared with the PEP, the risk of disease exacerbation was increased within 0–7 days after the first vaccination (IRR 7.71, 95% CI: 1.73–25.22, *p* = 0.002). However, this risk decreased within 7 days after the first vaccination. This result was an important complement to the knowledge of COVID-19 vaccination in MG patients and suggested that additional care may be required during the early phase after COVID-19 vaccination.

The primary outcome was robust in the subgroup analysis. Although the interaction effect did not reach statistical significance (*p* = 0.06), the risk of disease exacerbation was increased in patients ≥50 years during the ERP (IRR: 6.29, 95% CI: 1.54–30.67, *p* = 0.01). This result should be interpreted with caution and might be due to the small sample size and fewer disease exacerbations in different subgroups. The responsiveness of COVID-19 vaccines may be lower in the elderly due to their aging immune systems; consequently, they have fewer vaccine-related side effects (26, 27). Further larger-sample size studies are warranted to confirm these findings. Although no heterogeneity was found in different thymus status subgroups, it was noteworthy that most patients (4/5, 80%) with disease exacerbation in the ERP had combined thymoma, and three had a history of thymectomy. MG exacerbation is closely related to thymoma (28, 29). Although an increased risk after COVID-19 vaccination in thymoma-related MG has not been reported, we suggest that close attention should be focused on patients with thymoma-related MG after COVID-19 vaccination.

We set up the PRP to observe the impact of COVID-19 vaccination on the long-term disease exacerbation risk. Compared with the PEP, the rate of disease exacerbation in the PRP was not increased. At the end of the study, 43 (70.5%) patients reached MMS or better according to the MGFA-PIS, consistent with the PEP (42, 68.9%), suggesting that the COVID-19 vaccination has no long-term impact on disease relapses in MG patients.

There were 13 (21.3%) MG patients who had vaccine-related side effects after COVID-19 vaccination, including focal symptoms (pain, redness, and swelling at the injection site), fatigue, and fever. These vaccine-related side effects have been previously reported (23, 24), and the symptoms were mild and transient, further suggesting that COVID-19 vaccination is safe in MG patients.

### 4.1 Limitations

This study has several limitations: (1) given its retrospective nature, we cannot exclude the possibility that mild disease exacerbation was missed, leading to underestimating the risk of disease exacerbation after COVID-19 vaccination, especially in the PEP. This bias can be largely eliminated through data retrieved from prospective databases, in which patients’ medical information was recorded and preserved in detail in each regular follow-up; (2) although the data in our study were collected from two large MG research centers, the willingness concerning COVID-19 vaccination was very low in MG patients. The limited sample size may contribute to bias in the results, especially in subgroup analyses. However, we compensated for this weakness with the SCCS method, which can automatically control for time-invariant confounders and make the results more realistic; (3) only patients with stable disease status were enrolled in our study; thus, it is not clear to what extent our results generalize to those with unstable disease status. It is necessary to investigate the safety of the COVID-19 vaccine in these patients because vaccine hesitancy was greater in these patients.

## 5 Conclusion

Inactivated COVID-19 vaccines do not increase the risk of disease exacerbation in MG patients with stable disease status. A few patients experienced transient disease worsening, but the symptoms were mild. It is noted that more attention should be paid to thymoma-related MG, especially within 1 week after COVID-19 vaccination. In addition, COVID-19 vaccination has no long-term impact on disease relapse.

## Data availability statement

The raw data supporting the conclusions of this article will be made available by the authors, without undue reservation.

## Ethics statement

All patients signed the informed consent at the time of registry enrollment and agreed to the reuse of their data for additional studies. The study was approved by the ethics committees of the two research centers (K202207-10). Verbal informed consent was obtained from all patients; furthermore, patients who returned to the research center were requested to provide written informed consent.

## Author contributions

TC, Z-YL, ZR, C-BZ and S-SL contributed to the study design. ZR, XH and YS contributed to collecting and analyzing data. ZR, XH and YS contributed to the drafting of the manuscript. Y-LT, D-DM, D-LR, C-HL and S-JH contributed to collecting data. All authors contributed to the article and approved the submitted version.
